# MMP-2 and MMP-9 gene polymorphisms act as biological indicators for ulinastatin efficacy in patients with severe acute pancreatitis

**DOI:** 10.1097/MD.0000000000015831

**Published:** 2019-06-14

**Authors:** Lan Ling, Yan Li, Hong Li, Wen Li, Hong-Bo Zhang

**Affiliations:** a Emergency Department, China-Japan Friendship Hospital, Beijing; b Department of Vascular Surgery, The First Hospital of Jilin University, Changchun, P.R. China.

**Keywords:** efficacy, gene polymorphism, MMP-2, MMP-9, Rs243865, Rs3918242, severe acute pancreatitis, ulinastatin

## Abstract

**Background:**

Severe acute pancreatitis (SAP) is a severe form of inflammatory disease with a high mortality rate. Ulinastatin, as a urinary trypsin inhibitor (UTI), is a glycoprotein playing a critical role in SAP. Consequently, we identified the hypothesis that both matrix metalloproteinase-2 (MMP-2) and matrix metalloproteinase-9 (MMP-9) gene polymorphisms might promote the efficacy of ulinastatin in SAP.

**Methods:**

A total of 235 patients with SAP were treated by intravenous drip of ulinastatin for the duration of 10 days. Polymerase chain reaction-restriction fragment length polymorphism (PCR-RFLP) was performed for testing the distribution of genotypes and alleles frequency of MMP-2 and MMP-9 gene polymorphisms, and analyzing association of MMP-2 rs243865, MMP-2 rs2285053, MMP-9 rs3918242, or MMP-9 rs17576 with efficacy of ulinastatin in patients with SAP. Shesis software was adopted for analyzing single genotypes of MMP-2 and MMP-9 gene polymorphisms site A Generalized Multifactor Dimensionality Reduction (GMDR) model and a logistic regression analysis were used for analyzing effect of MMP-2 and MMP-9 gene polymorphisms on the efficacy of ulinastatin in treating patients with SAP.

**Results:**

CC genotype of MMP-2 gene rs243865 C>T was observed to have a better positive effect in promoting the efficacy of ulinastatin in comparison with CT and TT genotypes. Haplotype CCTG, CCTA, CTTG, and CTTA were combined by MMP-2 and MMP-9 gene polymorphisms which have the ability to increase the efficacy of ulinastatin in treating patients with SAP. MMP-2 gene rs243865 C>T site polymorphism was served as a favorable factor while the MMP-9 gene rs3918242 C>T site polymorphism was noticed as an unfavorable factor for the efficacy of ulinastatin in treating patients with SAP.

**Conclusion:**

The key findings clearly demonstrated that both the MMP-2 rs243865 and MMP-9 rs3918242 gene polymorphisms served as biological indicators for the efficacy of ulinastatin in treating patients with SAP.

## 1 Introduction

Acute pancreatitis (AP) as an inflammatory disease, occurs in the pancreas, and severe acute pancreatitis (SAP) was considered to be one of the types of AP with the abnormality named necrosis discovered in pancreas and in the adjacent connecting organs when inflammation predominantly responses to the cell impairment.^[1,2]^ However, about 15% to 20% of patients with SAP possess a high risk of mortality with systemic organ dysfunction or local pancreatic complications or both together.^[3]^ SAP is a common acute abdominal disease due to the swift complications and progression.^[4]^ In spite of the present treatment regimens, patients diagnosed with SAP still have a mortality rate of 10% to 20% owing to severe extra-pancreatic and pancreatic necrosis.^[5]^ Systemic inflammatory response syndrome in the early stage and multisystem (renal, respiratory, and cardiovascular) organ dysfunction in the later stage can be caused by a pro-inflammation in patients with SAP.^[6]^ Necrosis was considered to be one of the main risk factors for developing secondary infection, following appearance of a change from a pro-inflammatory action to an anti-inflammatory action and results in multiple organ failure.^[7]^ The prevention of SAP that results in the multiple organs failure should be prioritized predominantly during the treatment of SAP with both the comprehensive treatment and intensive care.^[8]^ Recently, ulinastatin has been widely utilized as one of the main ways of medication therapy to treat patients with SAP.^[9–11]^

Ulinastatin is a glycoprotein which can be obtained from human urine or applied pulmonary injury synthetically.^[12]^ Proteolytic enzymes including trypsin, elastase, and plasmin can be effectively inhibited by the ulinastatin and the hydrolases of lipase and amylase, thus relieving systemic inflammatory response so as to diminish the severity of pancreatitis.^[13]^ A study conducted by Lu et al have demonstrated that ulinastatin has the ability to suppress the excessive release of inflammatory regulators.^[14]^ Matrix metalloproteinases (MMPs) were reported to be a large group of calcium-dependent zinc with endo-peptidases.^[15]^ Created by various stromal and inflammatory corpuscles, MMPs are capable of decreasing extracellular matrix proteins and activating various bioactive molecules.^[16]^ It was previously demonstrated that cerulean-altered pancreatic MMPs (matrix metalloproteinase-2 [MMP-2], MMP-1, and matrix metalloproteinase-9 [MMP-9]) could induce pancreatitis in the early stage of inflammation, and might potentially influence the histological severity evaluation.^[17]^ Furthermore, the observed level of serum MMP-9 could be regarded as an efficacious indicator for assessing the SAP severity.^[18]^ Both MMP-9 and MMP-2 were considered as gelatinases, and activated MMP-2 and MMP-9 played vital roles in the regeneration and degradation of extracellular matrix mainly via their participation in matrix degradation and secretion into extracellular matrix.^[19]^

More importantly, single nucleotide polymorphisms (MMP-2 or MMP-9) have been identified and MMP-2 promoter polymorphisms rs243865 C>T and MMP-9 promoter polymorphism rs3918242 C>T have been identified as functionary factors in treating diseases.^[20–22]^ Also, the association of MMP-2 and MMP-9 gene polymorphism with ulinastatin efficacy in SAP patients has been previously reported.^[2]^ On the basis of investigations made by the influence of MMP-2 gene polymorphism and MMP-9 gene polymorphism on the efficacy of ulinastatin in patients with SAP, this study mainly aims to explore the correlation of MMP-2 and MMP-9 gene polymorphisms with the efficacy of ulinastatin in treating the patients with SAP.

## 2 Methods

### 2.1 Ethics statement

The study was approved by the Ethics Committee of China-Japan Friendship Hospital and all patients signed the informed consent.

### 2.2 Study subjects

A total number of 235 patients suffering from SAP admitted in China-Japan Friendship Hospital from January, 2011 to December, 2016 were selected as the study subjects, including 137 males and 98 females aged from 29 to 69 years old with an average age of 45.34 ± 5.66 years. All patients were diagnosed by B-ultrasound or computed tomography (CT) examination in accordance with the relevant diagnostic criteria established by the Pancreatic Surgery Group of Surgery Branch of Chinese Medical Association.^[23]^ The diagnostic criteria were mentioned as follows:

1) there was an external organ system involvement or organ failure;
2) local complications appeared.

The diagnosis of SAP will be confirmed if the examined people were presented with at least 3 of the following features: which are:

1) white blood cells >15 × 10^9^/L;
2) lactic acid detoxification >600 U/L;
3) glucose >180 mg/dL;
4) albumin <32 g/L;
5) urea nitrogen >16.065 mmol/L; 6) PaO_2_ <60 mmHg;
6) calcium <2 mg/L;
7) acute physiology;
8) chronic health evaluation (APACHE)-II score ≥8 points.

Patients

1) who were very sensitive to allergies;
2) who has history of allergy to drugs;
3) who were pregnant or lactating women;
4) who had received somatostatin treatment, gabexate or calcitonin were all excluded.

### 2.3 Polymerase chain reaction-restriction fragment length polymorphism (PCR-RFLP)

In the early morning, 5 mL of peripheral venous blood was extracted from all the patients who were observed to be in the state of fasting, and then the extracted blood was kept under the temperature of −4°C after using trisodium citrate for anticoagulation. DNA Extraction Kit (DP318 ∼03, Beijing Tiangen Biotechnology Co., Ltd., Beijing, China) was used in specific for extracting DNA fragments. PCR-RFLP was used to detect MMP-2 rs243865 C>T, rs2285053 C>T, and MMP-9 rs17576 A>G, rs3918242 C>T gene polymorphisms. Primers were designed by using the Primer Premier 5.0 software. The primer sequence was synthesized by Sangon Biotech Co., Ltd. (Shanghai, China). The primer sequence and length are shown in Table 1. A total of 20 μL PCR reaction system was composed of 100 ng of DNA fragments, the concentration of 2.4 μL of 10 × PCR buffer containing 15 nmol/L of MgCl_2_, 1.0 UTaq of DNA polymerase, 200 μmol/L of deoxyribonucleoside triphosphates (dNTPs) and 1 μL each of forward and reverse primer. PCR reaction conditions were as follows: pre-denaturation at 94°C for the duration of 5 minutes; 30 cycles at 94°C for 30 seconds, at 58°C for the duration of 45 seconds, and at 72°C for the duration of 45 seconds; final extension at 72°C for the duration of 10 minutes. Sterile double distilled water instead of template was used as negative control in each PCR reaction to ensure no contamination of the PCR reaction. The PCR product was digested and the reaction system (15 μL): 6 μL of PCR product and 1.5 μL of 10 × digestion buffer were added. Appropriate amount of sterile double distilled water was added to ensure that the total volume was 15 μL. After digestion in a water bath at the temperature of 37°C for 16 hours, the reaction was terminated. The product was performed on 3% agarose gel electrophoresis at 100 V for the duration of 15 minutes. Ethidium bromide (EB) staining and gel imaging system were used for determining the results. Positive control was established for each enzyme digestion reaction to ensure the accuracy of digestion.

**Table 1:**
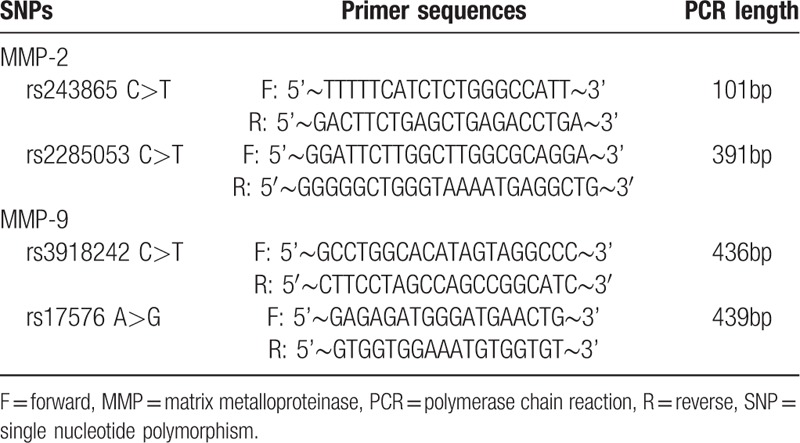
Primer sequences of MMP-2 and MMP-9 gene polymorphisms.

### 2.4 Treatment regimen and efficacy evaluation

All the patients diagnosed with SAP were continuously infused with ulinastatin through intravenous (No: 20080722; Guangdong Techpool Bio-pharma Co., Ltd., Guangdong, Guangzhou, China) at a dosage of 200,000 U ulinastatin + 250 mL 5% glucose liquid twice every day for 10 days long. Observation indicators: time of abdominal pain and distension, the total number of white blood cells in the peripheral blood (normal value: 7.3 × 10^9^–27.5 × 10^9^), blood amylase (normal value: 20∼100 U/L), urine amylase (normal value: 460 U/L), as well as the acute physiology and chronic health evaluation II (APACHE-II) improved score before and after treatment. APACHE-II score ^[24]^ includes 12 physiological scores, 1 age score, and 5 chronic disease scores. These 3 partial scores add up to the total points.

The main diagnosed symptoms and signs (including abdominal pain, abdominal distension) and the examined laboratory indicators (including white blood cells, blood amylase, and urine amylase content) were recorded on the 10th day before the treatment and at the beginning of treatment. The efficacy of drug included:

1) cure, on the treatment of the 10th day, abdominal pain of patients disappeared; the instrument test results showed that the laboratory indicators returned to normal physiological state;
2) effective, within the first 10 days of treatment, patients abdominal pain and abdominal distension of patients reduced; the laboratory indicators of more than 75% restored to the normal physiological state;
3) invalid, after the treatment, the mainly diagnosed symptoms and signs of patients did not show any improvement; the indicators of laboratory examination did not recover; some patients exhibited with the worst complications or died due to this disease. Total efficiency was the combination of cure rate and explicit efficiency.

### 2.5 Statistical analysis

All data were processed with the SPSS 21.0 software (IBM Corp., Armonk, NY), and Hardy–Weinberg Balance was used for detecting the population representation of genotype distribution. Comparison between the allele and genotype frequency was tested by χ^2^ test. Count data were expressed by rate or percentage or constituent ratio, and statistical analysis was carried out by chi square test. The measurement data were expressed with mean ± standard deviation, and the *t* test was used for comparison between groups. Analysis of variance (ANOVA) was used for comparison among multiple groups. Multiple factors were investigated by logistic regression and Shesis software was used to analyze the genotype of MMP-2 and MMP-9 gene site. *P* <.05 was considered significant statistically.

## 3 Results

### 3.1 Clinical outcomes of patients with SAP in the effective group and the ineffective group

As we observed, the total number of 235 cases of patients with SAP were treated with intravenous infusion of ulinastatin for 10 days long, and the total number of 189 cases were defined as effective and the total number of 46 cases were defined as ineffective according to the efficacy criteria. There were 109 males and 80 females included in the effective group with an age of 45.15 ± 4.92 years old, while there were 28 males and 18 females included in the ineffective group with an age of 46.15 ± 8.04 years old. Besides, there was no significant difference in the factors like age, sex, and body mass index (BMI) between the 2 groups (*P* >.05). By comparing the time of abdominal distention and abdominal pain disappearance after the treatment, leukocyte, urine amylase, blood amylase turns to normal physiological state, and APACHE-II score of these 2 groups, the results showed that the time of abdominal pain and abdominal distension, leukocyte, urine amylase and blood amylase recovery to normal physiological state in the effective group were significantly shorter than those in the ineffective group. Moreover, the APACHE-II score in the effective group was also lower in comparison with the ineffective group (all *P* <.05) (Table 2).

**Table 2:**
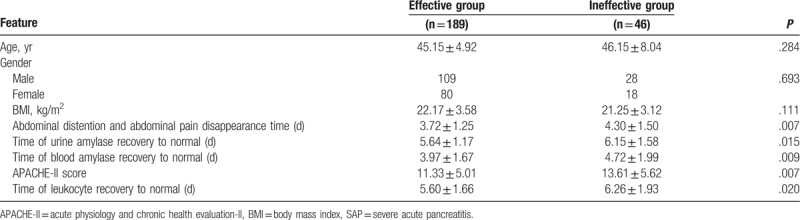
Clinical features of patients with SAP in the effective and ineffective groups.

### 3.2 Identification for the genotyping of MMP-2 (rs243865, rs2285053) and MMP-9 (rs3918242, rs17576) gene polymorphisms by PCR-RFLP

**Figure 1:**
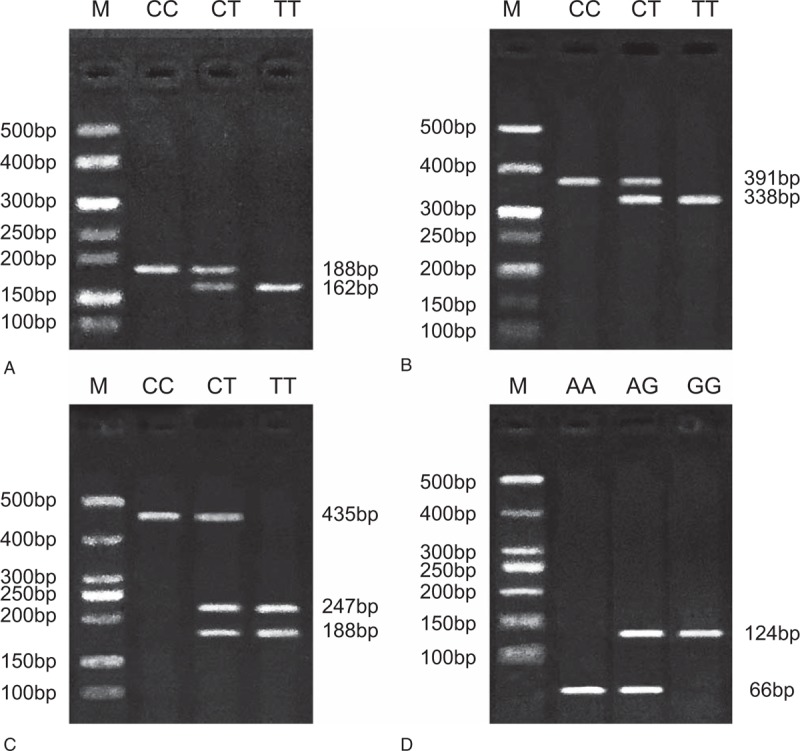
Genotypes of MMP-2 (rs243865, rs2285053) and MMP-9 (rs3918242, rs17576) gene polymorphisms detected by PCR-RFLP. A = rs243865 C>T site at MMP-2 gene, B = rs2285053 C>T site at MMP-2 gene, C = rs3918242 C>T site at MMP-9 gene, D = rs17576 A>G site at MMP-9 gene, MMP = matrix metalloproteinase, PCR-RFLP = polymerase chain reaction-restriction fragment length polymorphism.

PCR-RFLP was used for testing the distribution of different genotypes and alleles frequency of MMP-2 and MMP-9 gene polymorphisms. As the MMP-2 gene rs243865 C>T site PCR product was digested by Xsp I, the wild type CC band was 188 bp, heterozygous mutation TC band was 188 bp and 162 bp; homozygous mutant TT was only 162 bp after enzyme digestion (Fig. 1A). However, the PCR product of MMP-2 gene rs2285053 C>T site was digested by Hinf1, and a 391 bp band was found in wild-type CC. Three heterozygous mutation TC bands (391 bp, 338 bp, and 53 bp) existed after digestion, of which the 53 bp fragment was too small to be detected by the electrophoresis, so the genotypes were estimated by 338 bp and 391 bp bands. The homozygous mutant TT bands were 338 bp and 53 bp, respectively (Fig. 1B). Moreover, the PCR products of rs3918242 C>T site of MMP-9 gene were digested with Sph1, and the wild-type CC band was 435 bp; the heterozygous mutant CT bands were188 bp, 247 bp, and 435 bp; the homozygous mutant TT bands were 188 bp and 247 bp (Fig. 1C). The PCR product of rs17576 A>G site of MMP-9 gene was digested by MspI, and the wild-type AA bands were 66 bp and 58 bp. The heterozygous mutant AG bands were124 bp, 66 bp, and 58 bp. The 58 bp fragment could not be found in the electrophoretogram, thus the results could be deduced based on the 124 bp and 66 bp bands, and the homozygous mutant GG band was 124 bp (Fig. 1D). All above showed that sites of MMP-2 and MMP-9 gene polymorphisms was defined by genotyping.

### 3.3 The loci of MMP-2 and MMP-9 gene polymorphisms in SAP patients treated with ulinastatin

Hardy–Weinberg genetic balance was effective in testing the genotype frequency of all patients, and the obtained results showed that the genetic frequencies of all the genotypes reached required genetic balance, which was the indication of group representativeness (*P* >.05). In comparison with the wild homozygous CC, the heterozygous CT and mutant homozygous TT of rs243865 site in MMP-2 gene polymorphism were noticed to be significantly different between the ineffective and effective groups (all *P* <.05); significant differences were noticed in the distribution frequency of allele C and T between the ineffective and effective groups (*P* <.05); no significant differences were found in the distribution frequency of rs2285053 C>T site of MMP-2 gene polymorphism between the effective and ineffective groups (*P* >.05). Compared with the wild homozygous CC, the distribution frequency of mutation homozygous TT of rs3918242 site in MMP-9 gene polymorphism was noticed to be significantly different between the ineffective and effective groups (*P* <.05); no significant difference was observed in the distribution frequency of heterozygous CT between the ineffective and effective groups compared with wild homozygous CC (*P* >.05). Moreover, there was a significant difference in the distribution frequency of allele C and T between the ineffective and effective groups (*P* <.05); while no notable differences were found in the frequency of genotype rs17576 A>G between the effective and ineffective groups (*P* >.05) (Table 3).

**Table 3:**
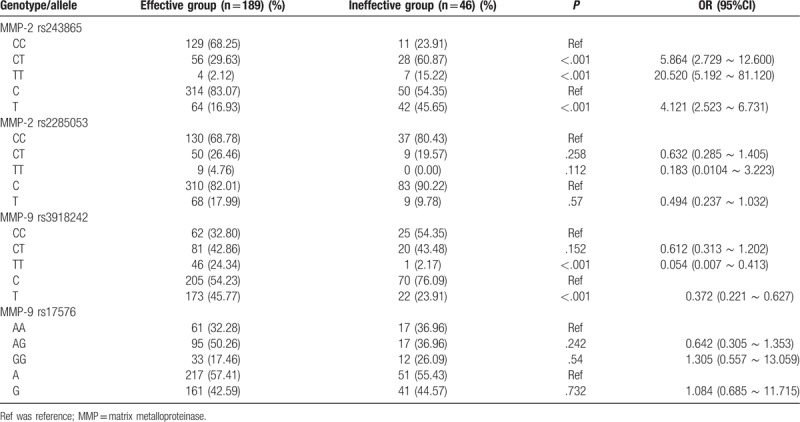
The genotype and allele frequencies of MMP-2 (rs243865, rs2285053) and MMP-9 (rs3918242, rs17576) polymorphisms in the effective group and ineffective group.

### 3.4 CC genotype rs243865 C>T site at MMP-2 supports ulinastatin in treating patients with SAP

The relationship between MMP-2 polymorphism or MMP-9 polymorphism and the efficacy of ulinastatin in treating patients with SAP was investigated. As the results showed in Table 4 and Table 5: after the 10 days of ulinastatin treatment in patients with SAP, the time duration of abdominal pain and distention, blood serum amylase, urine amylase and the efficient albumin recovery in CC genotype patients with rs243865 C>T site of MMP-2 gene polymorphism were all significantly shorter than those with CT and TT genotypes, but the APACHE-II improved score was significantly higher in comparison with CT and TT genotypes (all *P* <.05). Patients with TT genotype of rs3918242 C>T site MMP-9 gene polymorphism had significantly shorter time of abdominal pain and abdominal distension, and blood serum amylase, urine amylase and albumin recovery than those who with CC genotype, whereas the APACHE-II improved score was significantly higher in comparison with patients with CC genotype (all *P* <.05). However, there was no significant difference in time of abdominal pain, blood amylase, urinary amylase and albumin normalizing time and APACHE-II score of patients with CC genotype of rs2285053 C>T site carrying MMP-2 gene polymorphism and patients with CT and TT genotypes (all *P* >.05), while there was no significant difference in time of abdominal pain, blood amylase, urinary amylase and albumin normalizing time and APACHE-II score of patients with AA genotype of rs2285053 C>T site carrying MMP-2 gene polymorphism and patients with AG and GG genotypes (all *P* >.05). All these showed that patients with CC genotype rs243865 C>T site of MMP-2 gene polymorphism have better efficacy after treatment.

**Table 4:**
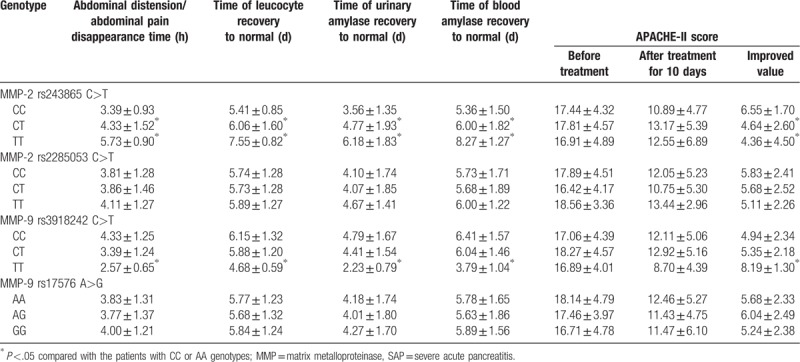
Association between MMP-2 polymorphism or MMP-9 polymorphism and the efficacy of ulinastatin in treating patients with SAP.

**Table 5:**
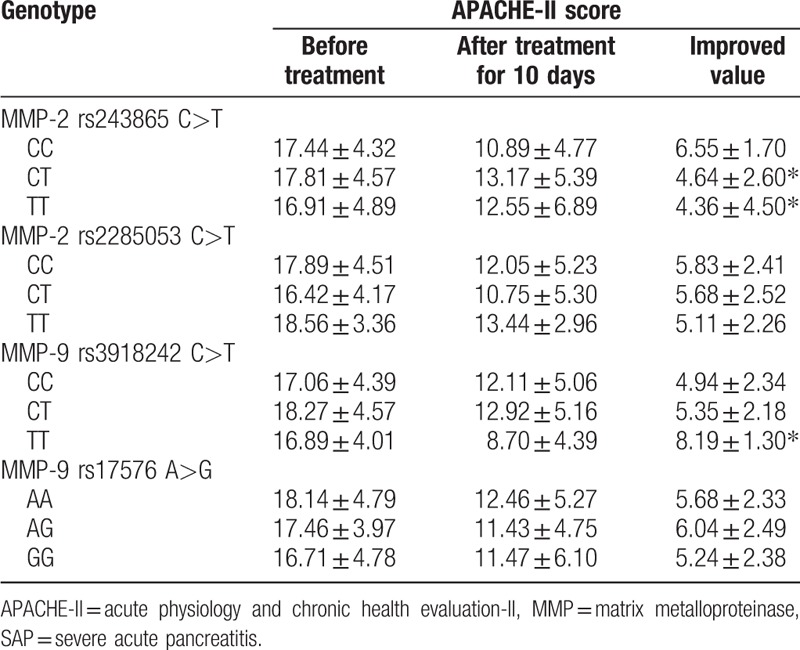
Association between MMP-2/MMP-9 polymorphisms and APACHE-II improved score of SAP patients before and after treatment with ulinastatin.

### 3.5 MMP-2 (rs243865/rs2285053) and MMP-9 (rs3918242/rs17576) polymorphisms in the efficacy of ulinastatin in treating patients with SAP

The linkage disequilibrium analysis of rs243865 C>T and rs2285053 C>T sites of MMP-2 gene polymorphism and rs3918242 C>T and rs17576 A>G sites of MMP-9 gene polymorphism was performed, and the results showed that there existed a strong linkage disequilibrium, and we could do haplotype analysis in this study. Thus, Shesis software was used for analyzing the haplotypes of rs243865 C>T and rs2285053 C>T sites of MMP-2 gene polymorphism and rs3918242 C>T and rs17576 A>G sites of MMP-9 gene polymorphism, in which genotypes with a frequency of less than 0.03 in each group should be discarded. As shown in Table 6, the haplotype CC and CT with rs243865 C>T and rs2285053 C>T sites of MMP-2 increased efficacy of ulinastatin in patients with SAP (OR = 0.501, 95% CI = 0.316–0.797, *P* = .003; OR = 0.138, 95% CI = 0.035–0.553, *P* = .001) and the haplotype TG and TA of MMP-9 rs3918242 C>T and rs17576 A>G sites increased efficacy of ulinastatin in patients with SAP (OR = 0.472, 95% CI = 0.229–0.976, *P* = .039; OR = 0.443, 95% CI = 0.235–0.836, *P* = .010). All these results predominantly indicated that the haplotype CC and CT of rs243865 C>T and rs2285053 C>T sites of MMP-2 gene polymorphism and the haplotype TG and TA rs3918242 C>T and rs17576 A>G sites of MMP-9 gene polymorphism has improved the efficacy of ulinastatin in patients with SAP.

**Table 6:**
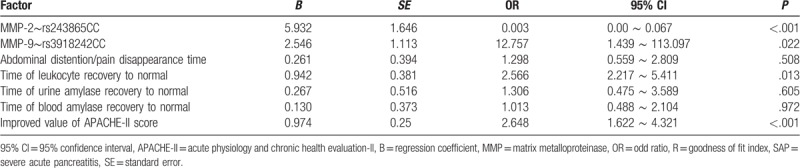
Multivariate logistic regression analysis suggests that MMP-2 gene rs243865 C>T site C allele is able to strengthen the efficacy of ulinastatin in treating patients with SAP.

### 3.6 MMP-2 gene rs243865 C>T site C allele improves the efficacy of ulinastatin in treating patients with SAP

The ulinastatin intravenous drip therapy was served as the dependent variable, the rs243865 genotypes of MMP-2 gene polymorphism, and the rs3918242 genotype of MMP-9 gene polymorphism, the disappearance time of abdominal pain and distension, blood amylase, urine amylase and leucocyte recovery time, and the APACHE-II improved score were served as efficient independent variables, which were included in the multivariate logistic regression analysis. The results in the Table 7 showed that the C allele on the MMP-2 gene polymorphism rs243865 C>T site was a favorable factor affecting the efficacy of ulinastatin in treating the patients with SAP (*P* <.05). However, the abnormal leucocyte recovery time and improved value of APACHE-II score of MMP-9 gene polymorphism rs3918242 C>T site allele C were the unfavorable factors of ulinastatin treatment in the patients suffering from SAP (*P* <.05). The results showed that not MMP-9 gene polymorphism rs3918242 C>T site C allele but MMP-2 gene polymorphism rs243865 C>T site C allele could improve the ulinastatin treatment.

## 4 Discussion

A previous study showed that gene polymorphisms might have potential effects on SAP treatment, thus we investigated the genotype and alleles among 235 patients with SAP to the synergistic effect of MMP-2 and MMP-9 gene polymorphisms on the efficacy of ulinastatin in patients suffer from SAP in this study.^[25]^ Consequently, our results showed that the synergistic effect of MMP-2 and MMP-9 gene polymorphisms might be the biological indicator for the efficacy of ulinastatin in patients with SAP.

SAP is different from mild AP due to the necrosis and hemorrhage of pancreatic tissues, as well as the life-threatening complications including electrolyte multiple organ dysfunction, imbalance, and inflammation.^[26]^ Ulinastatin functions as an acid-resistant trypsin inhibitor and works through stabilizing the lysosomal membranes and reducing organ dysfunctions, thereby treating the local complications resulted from SAP.^[27]^ By comparing the diagnosed symptoms of pre-treatment and post-treatment of patients with SAP, after the 10 days of ulinastatin treatment, the duration of diagnosed symptoms with MMP-2 gene polymorphism rs243865 C>T site CC genotype including abdominal distension and abdominal pain were noticed to be reduced significantly. Besides, albumin was significantly raised whereas the urine analysis, serum amylase and the APACHE-II score were markedly decreased. Consistently, previous studies noted that a reduction in the diagnosed symptoms and an elevation in the laboratory tests might attribute to the inhibition function of ulinastatin in the hydrolase enzyme activity of elastase, and trypsin.^[28,29]^ Therefore, this will result in improved tissue perfusion and microcirculation, inhibition of inflammatory mediator releasing and leukocyte activation. All these influences with ulinastatin could contribute to a decrease in the damage done to the human tissues and organs and a reduction in all kinds of complications.^[30]^

Importantly, the study is based on the hypothesis that MMP-2 and MMP-9 gene polymorphism might be correlated with the efficacy of ulinastatin in patients with SAP. As a collagenase type IV with the major function of degradation of type IV collagen, MMP-2 is a main component structured the basement membrane as well as extracellular matrix.^[31]^ MMP-9 is also a collagenase type IV and is found to accelerate the invasion and migration of inflammatory cells and vascular endothelial impairment, which is linked to a worsened inflammatory response.^[32,33]^ MMP-2 and MMP-9 have been identified to be closely associated with acute inflammatory reaction.^[34]^ Efficacy of ulinastatin could be improved by reducing the rate of acute respiratory distress syndrome, in which ulinastatin has the ability to decrease endotoxin absorption and inflammatory response to protect tissues and organs as well as to preserve physiological functions of tissues and organs by suppressing the hydrolytic enzyme release.^[35]^ A previous study indicated that ulinastatin could lower the plasma amylase levels and notably decrease the pathological changes in pancreas to diminish the risks of complications.^[11]^ It was also proved that ulinastatin could influence the protective roles played by MMP-9 and MMP-2 in tissues and organs.^[36]^

Moreover, our study predominantly demonstrates that the effect on MMP-2 rs243865 C>T CC genotype and MMP-9 rs3918242 C>T TT genotype were noticed to be more obvious in comparison with patients with other genotypes, which suggested that MMP-2 gene polymorphism and MMP-9 gene polymorphism are associated with the efficacy of ulinastatin in patients with SAP. Existed promoter factors including rs243865 C>T and rs3918242 C>T of gene polymorphisms of MMP-2 and MMP-9 might influence the gene transcription and lesions in gene function, respectively.^[37,38]^ The rs243865 C>T transition in MMP-2 gene enables to disrupt a Sp-1-binding site which contributes to a reduced MMP-2 promoter activity, while the transition of rs3918242 C>T in the MMP-9 gene has been proposed to be the cause of an increased promoter activity.^[39]^ Results of our study also indicate that the MMP-2 expression in the CC genotype was significantly increased compared with CT and TT genotypes, and individuals with the MMP-9 gene TT genotype had a higher risk consequence in comparison to the CC and CT genotypes. Furthermore, the previously study also noted that MMP-2 rs243865 C>T and MMP-9 rs3918242 C>T gene polymorphisms were closely related to cancer susceptibility.^[40,41]^

In conclusion, the present study found that the synergistic effect of MMP-2 gene polymorphism and MMP-9 gene polymorphism promoted the efficacy of ulinastatin in patients with SAP. However, larger sample sizes should also be included in the further studies to obtain more creditable results due to some limitations existing in the present study. Nevertheless, our findings provide evidence for clinical practice of ulinastatin treatment in patients with SAP.

## Acknowledgment

We would like to express our gratitude for the helpful comments received from our reviewers.

## Author contributions

L. Ling and Y. Li designed the study. L. Ling, H. Li, and W. Li collated the data, designed and developed the database, carried out data analyses and produced the initial draft of the manuscript. H.B. Zhang and Y. Li contributed to drafting the manuscript. All authors have read and approved the final submitted manuscript.

**Conceptualization:** Lan Ling, Yan Li.

**Data curation:** Yan Li.

**Methodology:** Lan Ling, Hong Li.

**Resources:** Hong Li.

**Supervision:** Wen Li.

**Writing – original draft:** Wen Li, Hong-Bo Zhang.

**Writing – review & editing:** Hong-Bo Zhang.
